# Supplementary material for: Splice-Junction-Based Mapping of Alternative Isoforms in the Human Proteome
Source: Cell Rep. Author manuscript; Available in PMC 2020 Jan 15. (PMC6961840; doi:10.1016/j.celrep.2019.11.026)
Supplement: 3 [file NIHMS1546469-supplement-3.zip › DF2/PXD0010154/Liver-47-Q5VWP3-YLEDNSDLFSEQLSHPMVAIPEHEALDSK.pdf]

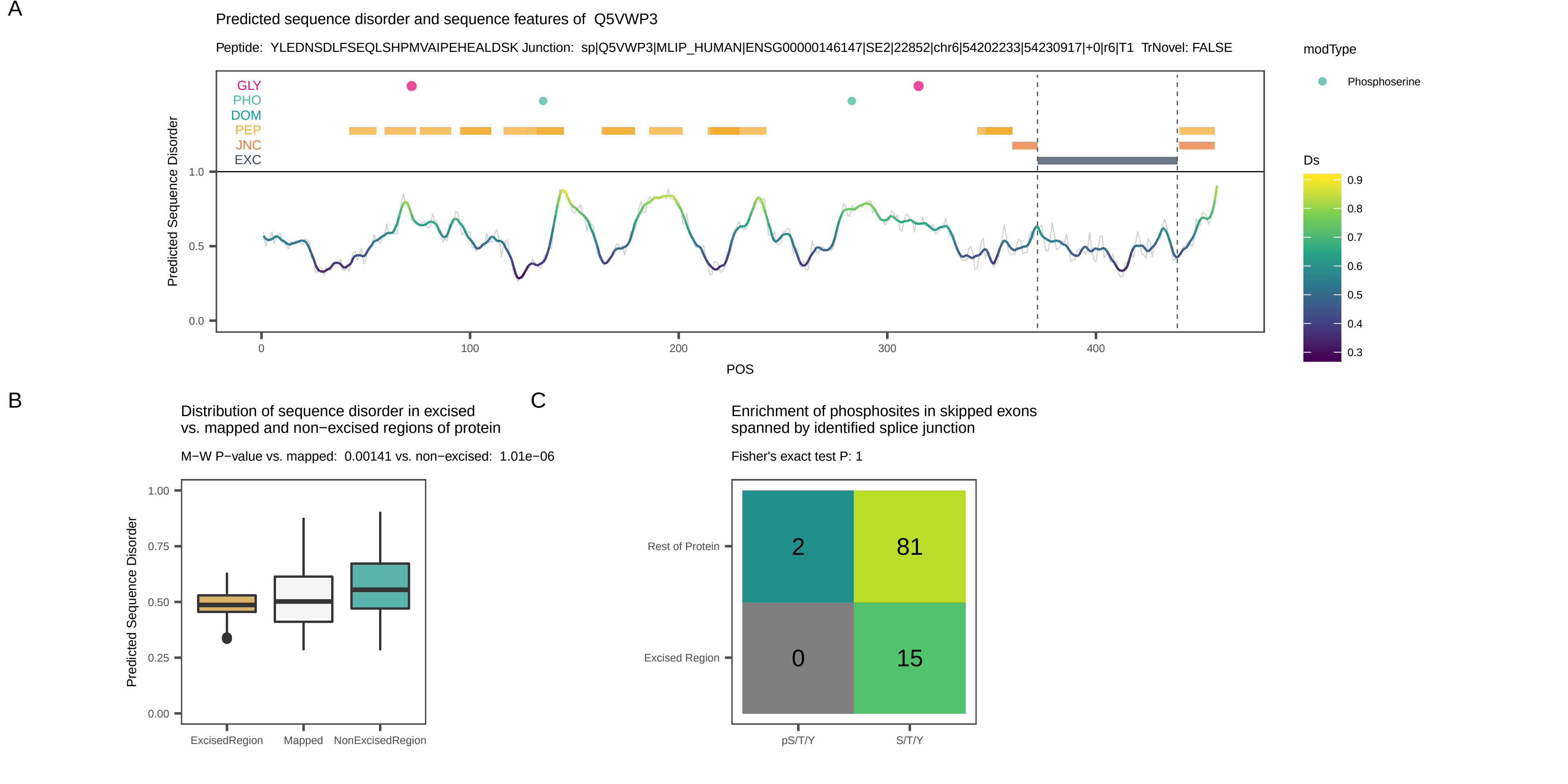
A
Predicted sequence disorder and sequence features of Q5VWP3
Peptide: YLEDNSDLFSEQLSHPMVAIPEHEALDSK Junction: sp|Q5VWP3|MLIP_HUMAN|ENSG00000146147|SE2|22852|chr6|54202233|54230917|+0|r6|T1 TrNovel: FALSE
GLY
PHO
DOM
PEP
JNC
EXC
Predicted Sequence Disorder
1.0
0.5
0.0
0
100
200
300
400
POS
modType
Phosphoserine
Ds
0.9
0.8
0.7
0.6
0.5
0.4
0.3
B
Distribution of sequence disorder in excised
vs. mapped and non−excised regions of protein
M−W P−value vs. mapped: 0.00141 vs. non−excised: 1.01e−06
Predicted Sequence Disorder
1.00
0.75
0.50
0.25
0.00
ExcisedRegion
Mapped
NonExcisedRegion
C
Enrichment of phosphosites in skipped exons
spanned by identified splice junction
Fisher's exact test P: 1
Rest of Protein
Excised Region
2
81
0
15
pS/T/Y
S/T/Y
